# Nitric oxide-an endogenous inhibitor of gastric acid secretion in isolated human gastric glands

**DOI:** 10.1186/1471-230X-4-16

**Published:** 2004-08-06

**Authors:** Anna Berg, Stefan Redeen, Ann-Charlott Ericson, Sven Erik Sjöstrand

**Affiliations:** 1 Department of Biomedicine and Surgery, Division of Cell Biology, Linköping University, Linköping, Sweden; 2 Surgery Department, University Hospital, Linköping, Sweden

## Abstract

**Background:**

Endothelial nitric oxide synthase (eNOS) has previously been detected in the glandular part of the human gastric mucosa. Furthermore, nitric oxide (NO) has been shown to influence gastric secretion in various animal models. The present study was conducted to investigate the influence of exogenously and endogenously derived NO on histamine- and cAMP-stimulated gastric acid secretion in isolated human oxyntic glands.

**Methods:**

Oxyntic glands were isolated from human gastric biopsies and were subsequently pre-treated with NO donors and nitric oxide synthase inhibitors and then exposed to histamine or dibutyryl-cAMP (db-cAMP). The secretory response of the glands was determined as accumulation of [^14^C]aminopyrine.

**Results:**

The histamine- or db-cAMP-induced acid secretion was attenuated by L-arginine, a known source of endogenous NO, and also by the NO-donors sodium nitroprusside (SNP) and S-nitroso-N-acetyl-penicillamine (SNAP). Pre-treatment with either of the NOS inhibitors N^G^-nitro-L-arginine methyl ester (L-NAME) or N^G^-nitro-L-arginine (L-NNA) enhanced the secretory response.

**Conclusion:**

Our results show that NO inhibits gastric acid secretion in isolated human gastric glands, and that there is endogenous formation of NO within the glandular epithelium in the vicinity of the parietal cells.

## Background

Nitric oxide (NO) is produced from L-arginine in a reaction catalyzed by the enzyme nitric oxide synthase (NOS)[1,2]. NO is an important biological signalling molecule that influences circulation by regulating vascular smooth muscle tone and modulating systemic blood pressure. Furthermore, NO is involved in neurotransmission; it is a critical factor in the inflammatory response and immunity [3-5]; and it has been shown to exert positive effects on mucosal defence in the gastrointestinal system. In several studies (for review, see [6]), chemically induced mucosal damage seemed to be reduced by simultaneous addition of NO and impaired by removal of NO from the gastric mucosa. An explanation for those findings might be that NO increases mucosal blood flow[7], and it has been suggested that NO augments the release of mucus[8]. It is likely that NO is also involved in the regulation of other secretory processes in the gastrointestinal system. Takeuchi and co-workers [9] have reported that NO inhibits the secretion of duodenal bicarbonate, whereas other investigators have proposed that bicarbonate secretion is stimulated by NO [10,11]. In addition, several studies have indicated that NO affects the secretion of gastric acid [12-16].

Animal experiments have provided conflicting information about the interaction between NO and gastric acid secretion. For instance, studies in vitro have shown that NO stimulates secretion of gastric acid in the mouse[17,18] and bullfrog[19]. In addition, similar results have been obtained in dogs [12]. However, other investigations have shown that NO inhibits gastric acid secretion in the rat [13,14], in gastric glands isolated from rabbits [15], and in mucosa from toads [16]. Studies of humans have provided data indicating that NO can both inhibit and augment intragastric pH [20,21], but it is not yet known how this compound participates in gastric acid secretion in humans.

In an earlier study, we found morphological support that endogenous NO plays a role in regulation of parietal cell function [22]. Also, the immunohistochemical data from that investigation revealed the presence of endogenous NOS in epithelial cells of the normal human oxyntic mucosa, more precisely, in both surface mucous cells and endocrine cells. In addition, we observed that there were close contacts between eNOS-positive cells and parietal cells either because the eNOS-positive cells contacted parietal cells via cytoplasmic processes or were invaginated by a parietal cell. Based on these findings, together with the chemical properties of NO, we concluded that NO derived from the endocrine-like cells might be a paracrine regulator of gastric acid secretion. In the present study, our aim was to verify the effect of exogenous NO on histamine- and cAMP-stimulated gastric acid secretion in humans, and also to determine whether endogenously derived NO has a functional effect on human parietal cells.

## Methods

### Subjects and ethical approval

Twenty-four healthy men ranging in age from 22 to 31 years were recruited as paid volunteers. The selection criteria stipulated that the subjects had to be free from disease and should not have taken any medications or imbibed alcohol for at least one week prior to examination. The men fasted for at least six hours before examination.

Pharyngeal anaesthesia was induced with lidocaine spray (Xylocain^®^, AstraZeneca, Södertälje, Sweden), after which routine gastroscopy was performed using an Olympus GIF-100 endoscope. Pinch biopsy forceps (Olympus FB 24K-1) were used to take tissue specimens from the greater curvature, immediately distal to the fundus. In all subjects, the gastric mucosa appeared to be normal, both macroscopically and histologically. All subjects tested negative for *Helicobacter pylori *infection in the urease breath test (Diabact^® ^UBT 50 mg ^13^C-urea, Diabact AB, Uppsala, Sweden).

The experimental procedures were approved by the Regional Ethics Committee for Human Research at University Hospital, Linköping, Sweden (File no. 02-039), and all subjects gave informed consent.

### Secretory study

#### Isolation and incubation of gastric glands

The current experiments were based on a technique that was first described in 1976 for use in rabbits in vitro [23] and is now well established for indirect determination of gastric acid secretion induced by various stimuli. The method of isolating gastric glands was initially developed for animal tissue, but it was later refined so that it could also be applied to small amounts of human tissue [24].

The human oxyntic mucosal biopsies used in our study were washed and stored no longer than 15 minutes in ice-cold oxygenated phosphate-buffered saline (PBS). The tissue specimens were cut into smaller pieces with a pair of scissors and transferred to oxygenated (100% O_2_) collagenase enzyme solution (130.0 mM NaCl, 12.0 mM NaHCO_3_, 3.0 mM Na_2_HPO_4_, 3.0 mM K_2_HPO_4_, 2.0 mM MgSO_4_, 1.0 mM CaCl_2_, 0.1 mM N^(alfa)^-tosyl-L-lysine chloromethyl ketone [TLCK], 10 μM indomethacin, 10 mM glucose, 2 mg/ml human serum albumin [HSA; Sigma], and 1 mg/ml collagenase type IA [Sigma]). The mixture was placed in a 37°C water bath and was gently stirred for 120 minutes, after which most of the treated specimen had disintegrated, leaving mainly isolated gastric glands. The mixture was subsequently filtered through a 200 μm mesh. The isolated glands were washed and re-suspended in pre-warmed (37°C) respiratory medium (132.4 mM NaCl, 1.0 mM NaH_2_PO_4_, 1.2 mM MgSO_4_, 5.4 mM KCl, 5.0 mM Na_2_HPO_4_, 1.0 mM CaCl_2_, 10 μM indomethacin, 10 mM glucose, and 2 mg/ml HSA). The glands were then transferred to vials containing fresh respiratory medium to which we added one of the following: the NOS inhibitor N^G^-nitro-L-arginine methyl ester (L-NAME; 1 mmol/L) or equivalent amounts of its biologically inactive enantiomer N^G^-nitro-D-arginine methyl ester (D-NAME); the NOS inhibitor N^G^-nitro-L-arginine (L-NNA; 0.1 mmol/L); either of the two NO donors sodium nitroprusside (SNP; 1 mmol/l) and S-nitroso-N-acetyl-penicillamine (SNAP; 0.1 mmol/L); the substrate for endogenous NO production, L-arginine (0.1 mmol/L) [15]. All gland suspensions, including those that were not stimulated, were incubated in a shaking water bath at 37°C for 30 minutes, after which we added histamine to a final concentration of 50 μmol/L or dibutyryl-cAMP (db-cAMP) to a final concentration of 1 mmol/L. To prevent degradation of cyclic nucleotides, we added 0.1 mmol/L 3-isobutyl-1-methylxantine (IBMX) to all stimulations.

#### Determination of the [^14^C]aminopyrine accumulation ratio

A well-established method used to indirectly measure acid secretion by isolated gastric glands is to determine accumulation of ^14^C-labeled aminopyrine (AP) in the glands themselves and in the supernatant after centrifugation and then calculate the ratio between those two values (called the AP ratio) [23]. In short, acid secretion was stimulated at 37°C for 40 minutes, and after that 0.5 μCi [^14^C]labeled aminopyrine was added to the vials, which were then further incubated at 37°C for 90 minutes. Thereafter, the gland suspension was transferred to previously dried and weighed tubes, which were centrifuged at 4,000 rpm for two minutes. The supernatant was removed and transferred to scintillation vials. The pellets (glands) were dried at 100°C, and the dry weight was determined, and the glands were subsequently re-suspended in 0.5 mol/L NaOH at 60°C and transferred to scintillation vials. The radioactivity of the glands and the supernatant was determined in a liquid scintillation counter (1214 Rackbeta, LKB), and the AP ratio was calculated using the following formula[24]:



where IGW is intraglandular water volume (= 2 × the dry weight of glands in mg).

Background accumulation of AP is included in the values representing the secretory response. The AP ratios for background and histamine-and db-cAMP-stimulated conditions differed between the individuals. Therefore, for each subject, we determined the AP ratio for stimulated glands and considered that value to be 100% and used it as an individual reference value. All values are based on single analyses.

### Statistics

Data were analysed by one-sample sign tests comparing median values using MINITAB™ Statistical Software. P values less than 0.05 were considered significant.

### Immunohistochemistry

Isolated gastric glands from five test subjects were placed on charged Super Frost*/Plus glass slides (Menzel-Gläser, Germany) and then washed with PBS and permeabilized with 100% ethanol at -70°C for 5 min. Thereafter, the slides were incubated at room temperature overnight with rabbit anti-NOS3 antibody (1:1000; Santa Cruz Biochemicals) and then washed thoroughly in PBS and incubated for 1 h with biotinylated goat anti-rabbit secondary antibody. Slides where primary antibody had been left out served as negative controls. The slides were subsequently washed again, and biotinylated antibody was detected by exposure to 20 μg/ml Texas Red^® ^Avidin (Vector Laboratories) for 1 h. Following that treatment, the slides were washed and coverslipped using Vectashield^® ^mounting medium. A Nikon Eclipse^® ^E800 fluorescence microscope with a VFM EPI-fluorescence attachment was used to examine and evaluate the slides. A band-pass filter with a wavelength range of 520–560 nm and a long-pass filter with cut-on wavelength at 590 nm (for emitted light) were employed to visualize the Texas Red^® ^molecules.

### Hematoxylin and eosin staining

For morphological evaluation, glands were fixed on glass slides and stained with Harris hematoxylin for five minutes and 0.5% eosin Y for two minutes. Each step was followed by a rinse in tap water.

## Results

**Figure 1:**
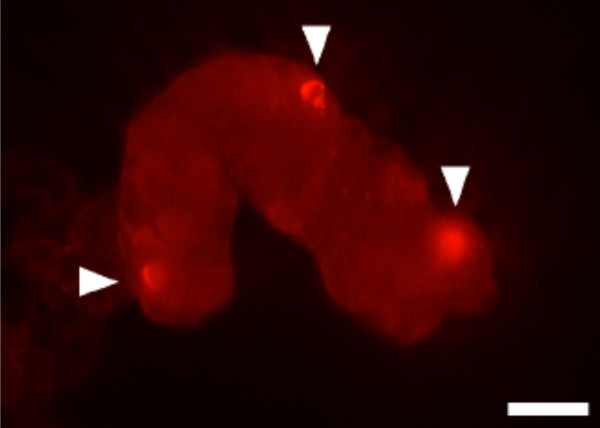
**Immunofluorescence of an isolated gastric gland. **Immunolocalization of eNOS (arrowheads) in a gastric gland isolated from human oxyntic mucosa was achieved using a rabbit anti-eNOS polyclonal antibody. The results were visualized with Texas Red^®^-conjugated goat anti-rabbit IgG. Bar = 30 μm.

We studied the effects of NO on acid secretion induced by various stimulants in gastric glands isolated from stomach biopsies from human. Morphological examination of the hematoxylin-eosin-stained slides revealed that the isolation procedure had successfully yielded whole-gland preparations and that parietal cells were present in the gastric glands. Immunohistochemical analysis showed that the isolated glands contained eNOS-immunoreactive cells (Fig. 1), which agrees with results obtained using other types of mucosal preparations [22]. Control experiments were primary antibody was excluded showed no immunoreactivity.

### Background AP-accumulation

**Figure 2:**
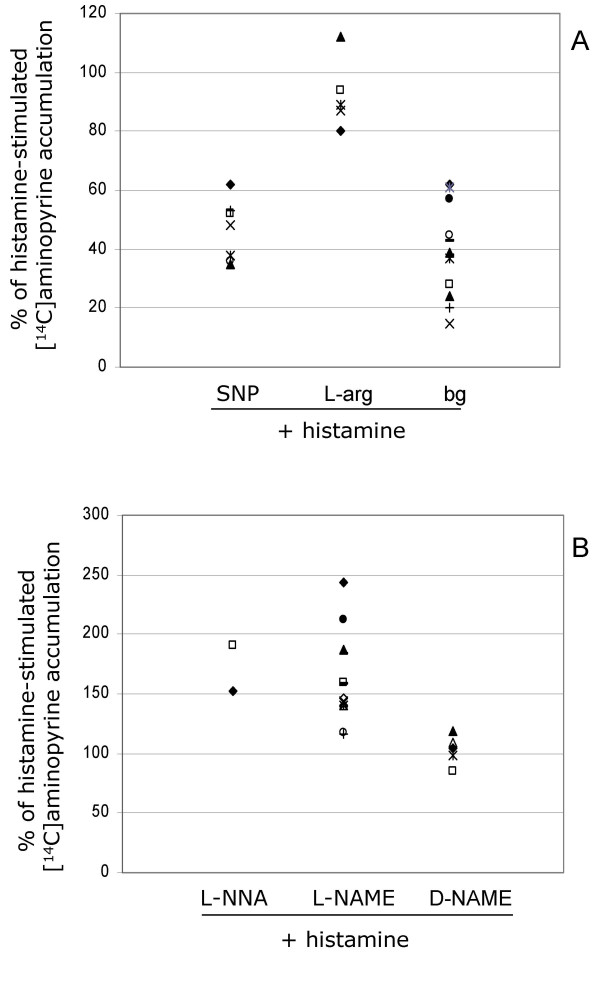
**Accumulation of ^14^C-labeled aminopyrine in histamine-stimulated gastric glands. **All values are expressed as percent of the gastric acid secretion induced by histamine (considered to be 100%), which was calculated separately for gastric glands isolated from each of the healthy volunteers. Each symbol represents the results for one individual. a) Accumulation of ^14^C-aminopyrine in glands pretreated with the NO donor sodium nitroprusside (SNP, 1 mmol/L) or with L-arginine (0.1 mmol/L), the substrate for endogenous NO production. It can be seen that SNP markedly reduced AP accumulation (median = 48%; p < 0.05), which indicates that NO inhibits acid secretion from the isolated glands. Background ^14^C-aminopyrine accumulation (bg) is also shown. b) Accumulation of ^14^C-aminopyrine in gastric glands pretreated with the NOS inhibitors L-NNA (0.1 mmol/L) and L-NAME (1 mmol/L), respectively. L-NAME caused increased accumulation (median = 147%; p < 0.05), which suggests that acid secretion is elevated when endogenous NO production is prevented, indicating an inhibitory role for endogenous NO in human gastric glands. D-NAME, which is the biologically inactive stereo isomer of L-NAME, did not have an effect on acid secretion, and it was therefore used as a control substance.

**Figure 3:**
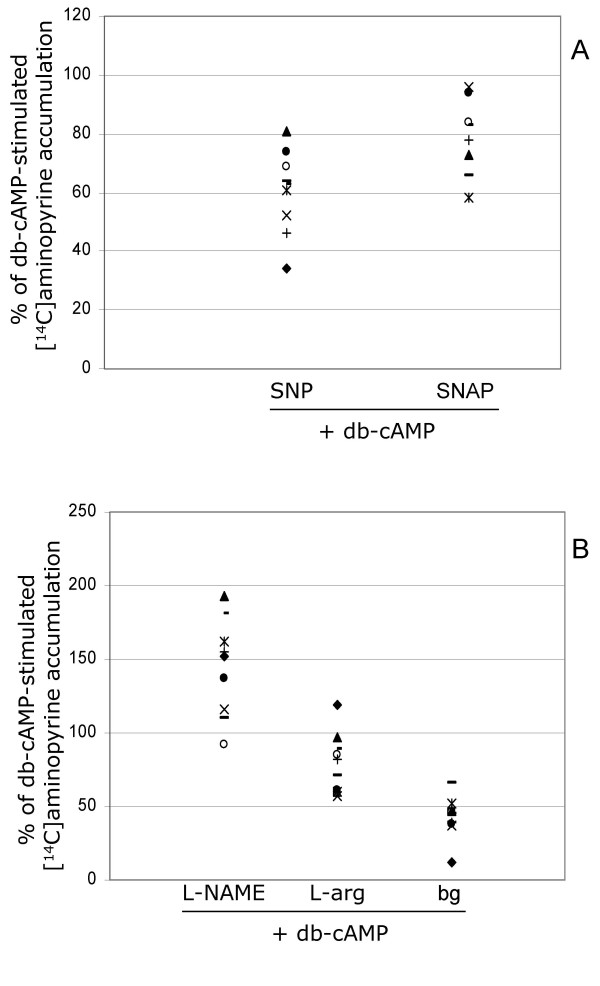
**Accumulation of ^14^C-labeled aminopyrine in db-cAMP-stimulated gastric glands. **All values are expressed as percent of a value representing the gastric acid secretion induced by db-cAMP (considered to be 100%), which was calculated separately for each of the studied subjects. Each symbol represents the data for one individual. a) Pretreatment with SNP (median = 63%; p < 0.05) or SNAP (median = 81%; p < 0.05) to release exogenous NO before adding db-cAMP to stimulate acid secretion reduced the accumulation of ^14^C-aminopyrine in gastric glands, as compared to levels of secretion seen in untreated glands. This indicates that NO can inhibit acid secretion in gastric glands isolated from humans. b) Treatment with L-NAME to inhibit NOS in the gastric glands increased the accumulation of ^14^C-aminopyrine after stimulation with db-cAMP (median = 152%; p < 0.05). Those results indicate that NO inhibits db-cAMP-induced acid secretion. The NO substrate L-arginine reduced the accumulation of ^14^C-aminopyrine in db-cAMP-stimulated glands (median = 77%; p < 0.05). Background accumulation (bg) is also shown.

Background AP accumulation was observed in the isolated glands, with a median AP ratio of 8.6 (range 2.5–22.1; n = 19). After administration of 50 μmol/L of histamine or 1 mmol/L of db-cAMP, the median AP ratios were 24.7 (5.8–64.5; n = 16) and 38.2 (range 7.6–47.8; n = 11) respectively. The response to both histamine and db-cAMP exceeded the background by a factor of about 2–4 in all preparations (Figs. 2 and 3).

### Effect of NO donors and NOS inhibitors on histamine-stimulated gastric acid secretion

Pre-treatment of isolated glands with the exogenous NO donor SNP (1 mmol/L) reduced the histamine response to a median of 48% (n = 7) of the response seen in non-pre-treated glands (100%). In four out of five gland preparations, the substrate for endogenous NO formation, L-arginine (0.1 mmol/L), decreased the AP ratio. This effect however was not statistically significant (Fig. 2a). When isolated glands were pre-treated with NOS inhibitor L-NAME (1 mmol/L), and then exposed to histamine, the AP ratio was markedly elevated to a median value of 147% (n = 13). Although a small number of individuals were tested, L-NNA (0.1 mmol/L) yielded a similar result; 172% (n = 2). By comparison, in control experiments, the L-NAME analogue D-NAME (1 mmol/L) had no effect at all on histamine-stimulated acid secretion (Fig. 2b).

### Effect of NO donors and NOS inhibitors on db-cAMP-stimulated gastric acid secretion

Exposing the isolated glands to db-cAMP increased the secretion of gastric acid compared to the background level. Compared to untreated glands, those that were pre-treated with SNP (1 mmol/L) or SNAP (0.1 mmol/L) accumulated less AP after stimulation with db-cAMP; 63%(n = 9) and 81%(n = 8) respectively (Fig. 3a). Moreover, similar to histamine-stimulated secretion, the db-cAMP-induced secretion was increased to a median of 152% (n = 9) in glands that had been pre-treated with L-NAME (1 mmol/L). Although no effect on L-arginine on histamine-stimulated acid secretion could be seen, there was a significant effect of L-arginine on db-cAMP-induced secretion. Acid output was inhibited to a median of 77% (n = 10) in those pre-treated with L-arginine (0.1 mmol/L)(Fig. 3b).

## Discussion

The method of using isolated gastric glands in vitro is well suited for studying the interaction between gastric acid secretion and various endocrine signals. This technique has been thoroughly evaluated, chiefly in experiments on animals, and it has been reported to offer good reproducibility [23]. Furthermore, in a study of gastric glands isolated from stomach biopsies taken from humans [24], it was found that both histamine and db-cAMP induced secretory responses that were reproducible when repeated using gland preparations from the same subject, although there was considerable interindividual variation. Fellenius et al. [24] and Haglund et al. [25] have reported that both histamine and db-cAMP stimulated gastric acid secretion from isolated human gastric glands, and this was observed as a two- to threefold increase in AP accumulation compared to the background level. Those results agree with our data obtained using histamine and db-cAMP. It is known that SNP releases NO [26], and in our experiments SNP reduced secretion of gastric acid from isolated glands. Hence, NO inhibits acid secretion in isolated human gastric glands. Some of the effects of SNP may be due to cytotoxic interactions, although no such impact was found in a study of isolated rabbit gastric glands [15]. To rule out the possibility of a cytotoxic influence, we performed complementary experiments using the NO donor SNAP, which has chemical properties that differ from those of SNP. In those experiments, we observed the same reduction in AP accumulation after stimulation with db-cAMP, which further favours the conclusion that NO is in fact responsible for observed results. In db-cAMP-stimulated glands, the induced secretory response was reduced by L-arginine, a compound that depends on an endogenous factor (i.e., eNOS) to generate NO, although that reduction was not as pronounced as the decrease induced by SNP and SNAP. There are a number of possible explanations for that observation. If there was already enough L-arginine in the glands to sustain NO production at the time of the experiment, addition of L-arginine was therefore without effect. Furthermore, L-arginine may have had a weak impact because the effect of NO occurred through up- or down regulation of the enzyme NOS and was not influenced by access to substrate. Notwithstanding, L-arginine did decrease the accumulation of AP, albeit not as much as the exogenous NO donors did at the present doses. This indicates that some specific process is responsible for generating NO from L-arginine in isolated gastric glands. These results are consistent with studies showing that SNP, SNAP, and L-arginine inhibited histamine-stimulated acid secretion from gastric glands isolated from animals [13,15]. L-arginine has also been observed to reduce carbachol-stimulated acid secretion in toads [16].

In a previous study conducted by our research group [22], examination of the glandular epithelium of specimens of human oxyntic mucosa revealed that one particular type of cells contained NOS. These eNOS-immunoreactive cells, defined as endocrine cells, were in close contact with parietal cells. These two characteristics suggest that cells of this type release NO, which thus might be a paracrine regulator that directly affects the function of parietal cells. That assumption may be supported by a number of other conditions. For example, NO can easily penetrate cell membranes, which may indicate an intracellular site of action. Also, NO has a rather short life span, which implies that sources needed to generate this oxide must be available close to the NO target cell. In this study, the occurrence of eNOS in the glands is shown, but earlier extensive investigations using antibodies against both nNOS and iNOS have not revealed presence of any of the two isoforms in the glandular epithelium of normal human subjects (unpublished observation). Similar to results obtained in a study of isolated rabbit gastric glands[15], we found that the NOS inhibitors L-NAME and L-NNA, but not D-NAME, amplified the secretion-stimulating effect of histamine, which further indicates that the isolated human glands we used contained the enzyme NOS. Both the increase in the AP ratio that we observed following inhibition of NOS and the decrease in secretory responses that we noted in glands treated with L-arginine strengthen the hypothesis that NO is produced by cells in the glandular epithelium and, when released, it interferes with stimulated acid secretion. Interestingly, the mentioned observations suggest that the release of NO is sustained, regardless of whether acid secretion is stimulated, which implies that NO functions as an endogenous inhibitor of gastric acid secretion. The intensity of this inhibition probably depends on the number of eNOS-containing endocrine-like cells that are present in the vicinity of the parietal cells.

The exact mechanisms behind this paracrine regulation of gastric acid secretion is yet to be elucidated. There are several different pathways within the parietal cell that might be affected by NO. It can induce ADP ribosylation of G-actin [27], thereby influencing the cytoskeleton. This could be essential for the morphological changes that parietal cells exhibit during acid secretion. Accordingly, if NO does have a persistent impact on an element such as the cytoskeleton, it might play a role in the membrane recycling hypothesis proposed by Forte et al. [28]. Briefly, that theory suggests that parietal cells undergo the following morphological alterations: they have a large active secretory surface during the stimulatory phase, and they display a minimal active secretory surface during the resting phase.

Since it is known that guanylate cyclase is a general target of NO in many cell systems, some investigators have suggested that NO exerts its effects via cyclic guanosine 3',5'-monophosphate (cGMP) in both rat and rabbit parietal cells [13,15]. An ongoing study in our laboratory will show whether this is the case in human parietal cells. Downstream effects of cGMP may include activation of a number of effectors, such as ion channels, protein kinases, and phosphodiesterases [29]. It is also plausible that NO can exert its effect alone, without acting through other signalling molecules. Under experimental conditions, NO can induce nitrosylation and nitration of cellular proteins, although that is probably not the case in vivo, since those two processes often result permanent damage to vital functions [30]. There is a possibility that the suppression of acid secretion occurs not only at parietal cell level, but via other cell types. ECL-cells are probably present in the glandular preparation and in the rat, these cells have the ability to release histamine in response to increased intracellular levels of cAMP [31]. NO can inhibit this histamine-release [14] and thereby further contribute to the inhibition of acid secretion. Although there is little known about human ECL-cells and the effects of NO on histamine-release there are studies that indicate species differences in other histamine-secreting cells. For example, rat mast cells have been shown to produce an "NO-like factor" which inhibits histamine-release [32] while there are investigations that indicate that NO does not affect histamine-secretion in human basophils [33]. At present we can only establish differences in inhibitory response to l-arginine for histamine and db-cAMP stimulation.

Further investigations are needed to clarify the role of NO in parietal cell function.

## Conclusions

The findings of the present study suggest that NO produced endogenously in the human oxyntic mucosa can reduce the stimulatory effects of histamine or db-cAMP on gastric acid secretion. We obtained uniform results for gastric glands isolated from different healthy human subjects, which implies that NO released from specific cells within the secretory mucosa plays an important physiological role in the regulation of gastric acid secretion.

## Competing interests

None declared.

## Authors' contributions

AB participated in the design and coordination of the study, performed the secretory studies, carried out the immunohistochemical procedures, and drafted the manuscript. SR was the surgeon in charge and carried out all gastroscopic procedures. ACE and SES were involved in the design of the study and in drafting of the manuscript. All authors read and approved the final manuscript.

## Pre-publication history

The pre-publication history for this paper can be accessed here:


